# Realizing active targeting in cancer nanomedicine with ultrasmall nanoparticles

**DOI:** 10.3762/bjnano.15.98

**Published:** 2024-09-30

**Authors:** André F Lima, Giselle Z Justo, Alioscka A Sousa

**Affiliations:** 1 Department of Biochemistry, Federal University of São Paulo, São Paulo, SP 04044-020, Brazil

**Keywords:** active targeting, cancer, nanoclusters, renal clearance, ultrasmall nanoparticles

## Abstract

Ultrasmall nanoparticles (usNPs) have emerged as promising theranostic tools in cancer nanomedicine. With sizes comparable to globular proteins, usNPs exhibit unique physicochemical properties and physiological behavior distinct from larger particles, including lack of protein corona formation, efficient renal clearance, and reduced recognition and sequestration by the reticuloendothelial system. In cancer treatment, usNPs demonstrate favorable tumor penetration and intratumoral diffusion. Active targeting strategies, incorporating ligands for specific tumor receptor binding, serve to further enhance usNP tumor selectivity and therapeutic performance. Numerous preclinical studies have already demonstrated the potential of actively targeted usNPs, revealing increased tumor accumulation and retention compared to non-targeted counterparts. In this review, we explore actively targeted inorganic usNPs, highlighting their biological properties and behavior, along with applications in both preclinical and clinical settings.

## Review

### 1 Introduction

Nanotechnology has opened new avenues for tackling unmet challenges in medicine [1–3]. In the field of oncology, a notable application involves the use of engineered nanoparticles (NPs) designed to transport therapeutic agents with precise delivery to tumor sites. This approach aims to mitigate toxic effects associated with off-target drug delivery and optimize therapeutic efficacy.

For decades, the enhanced permeability and retention (EPR) effect has stood as the central mechanism for driving passive NP delivery to tumors [4–5]. In this model, leaky blood vessels and a compromised lymphatic drainage system contribute to the preferential NP extravasation and accumulation within solid tumors. However, recent evidence challenges this paradigm, suggesting that NP extravasation into tumors primarily occurs via transendothelial transport pathways [6–7]. Regardless of the mode of NP extravasation, active targeting strategies have been widely explored to further enhance NP accumulation in tumors and NP internalization by cancer cells [8–9]. Active targeting involves the modification of NPs with targeting ligands (i.e., small molecules, peptides, or antibodies) that bind to overexpressed receptors within the tumor microenvironment.

Despite the promise of nanomedicine, neither passive nor active delivery strategies have significantly improved clinical therapeutic outcomes for solid tumors [10–12]. Reasons for the poor clinical performance of passive tumor targeting are the considerable heterogeneity of the EPR effect in humans, alongside the restricted diffusion of NPs across the dense tumor stroma [4–5,13–14]. Reasons for the limited performance of active targeting include its reliance on passive targeting, the more complex designs of targeted NPs, the potential for attached functional ligands to increase phagocytic capture and shorten blood circulation time, and the formation of a protein corona that may block the targeting ligand on the particle surface [15–17].

**Figure 1:**
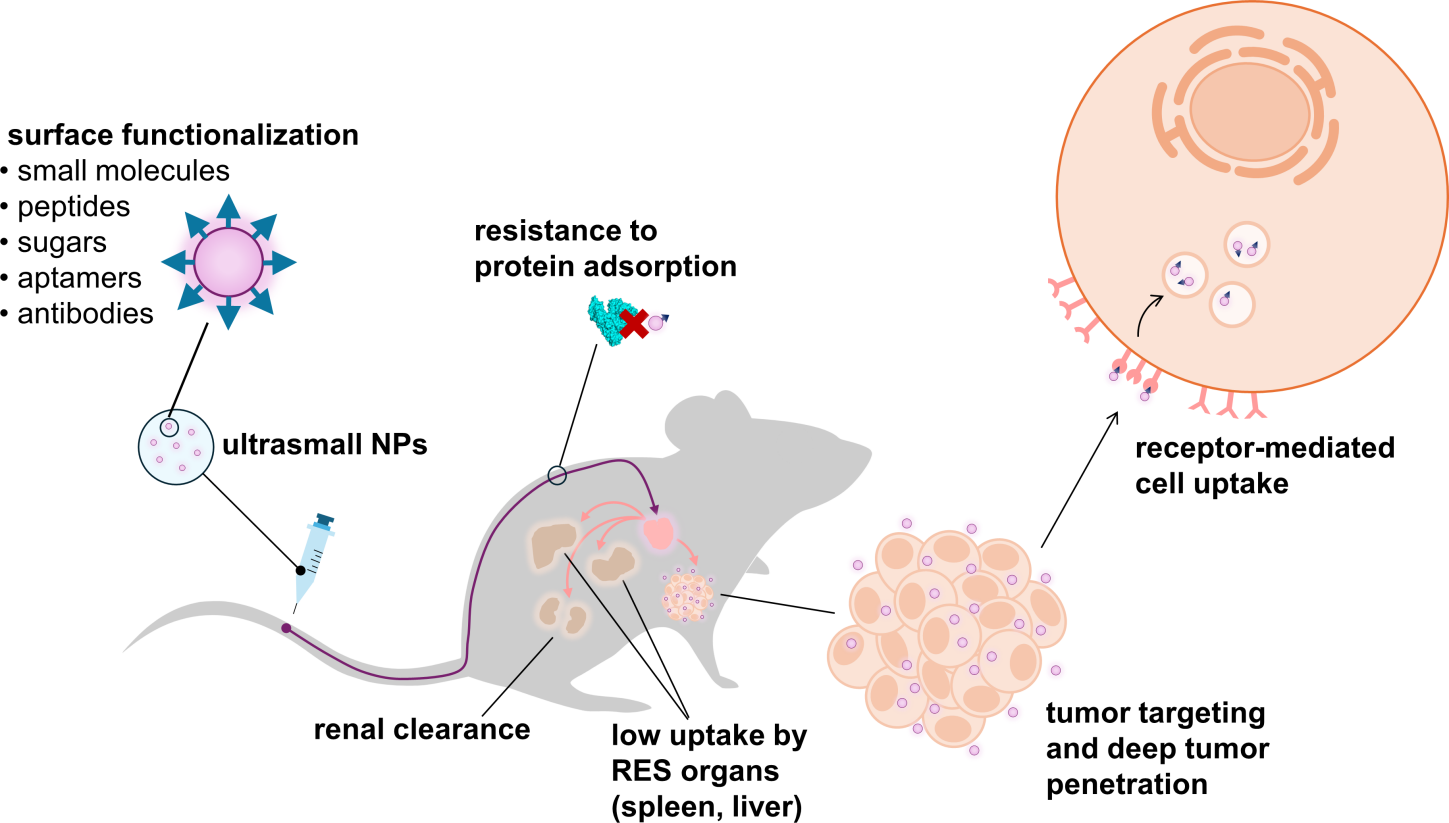
Schematic representation of ultrasmall nanoparticles, highlighting their unique biological functionalities and physiological behavior. The figure was created using PowerPoint software, incorporating a graphical illustration (mouse outline) adapted from https://www.phylopic.org, licensed under CC0 1.0 (https://creativecommons.org/publicdomain/zero/1.0).

Over the last decade, a special class of inorganic NPs, termed ultrasmall NPs (usNPs), has attracted increased attention in the field of cancer nanomedicine [18–24]. This increased focus is attributed to their unique physicochemical properties, biological functionalities, and physiological behavior, collectively addressing limitations associated with conventional large NPs (Figure 1).

In this topical review, we begin by defining inorganic usNPs, highlighting their importance in cancer nanomedicine, and discussing the implementation of active targeting strategies. Then, we explore various modalities of actively targeted usNPs and their current applications in cancer diagnosis and treatment.

### 2 Inorganic ultrasmall NPs in cancer nanomedicine

Important classes of inorganic usNPs under investigation for cancer nanomedicine include metallic usNPs (gold, silver), oxide and sulfide usNPs (silica, iron oxide, copper sulfide), and rare earth-based usNPs (cerium oxide, gadolinium oxide) [22]. Ultrasmall NPs have dimensions comparable to those of a typical globular protein of 3 to 6 nm in diameter [22,25], although the precise size criteria can vary among researchers. For the purpose of this discussion, usNPs are defined as being small enough to undergo renal clearance. While this usually entails usNPs smaller than the kidney filtration barrier of 5–6 nm [26–28], slightly larger particles have also been found to undergo renal excretion in some cases.

Ultrasmall NPs are situated at the interface between small molecules and conventional NPs, and so they provide a unique opportunity to leverage distinctive properties inherent to both domains [25,29–30]. On one hand, usNPs and their conjugates can behave as biomolecules in terms of biomolecular interactions and physiological behavior [31–35]. Additionally, certain types of usNPs, especially gold nanoclusters (AuNCs), manifest molecule-like physical and chemical properties, such as luminescence [36–37]. Simultaneously, usNPs – whether used alone or conjugated to drugs, diagnostic probes, and targeting ligands – can function as a more conventional NP platform in nanomedicine applications [18,21–22,38–40]. In diagnostic applications, usNPs have been employed in diverse imaging modalities, including optical imaging [41–42], X-ray computer tomography [43], photoacoustic imaging [41,44], magnetic resonance imaging [20,40], and positron emission tomography [45–46]. In therapeutic applications, usNPs have been used for drug delivery as well as served as phototherapeutic agents and radiosensitizers [47–51].

**Figure 2:**
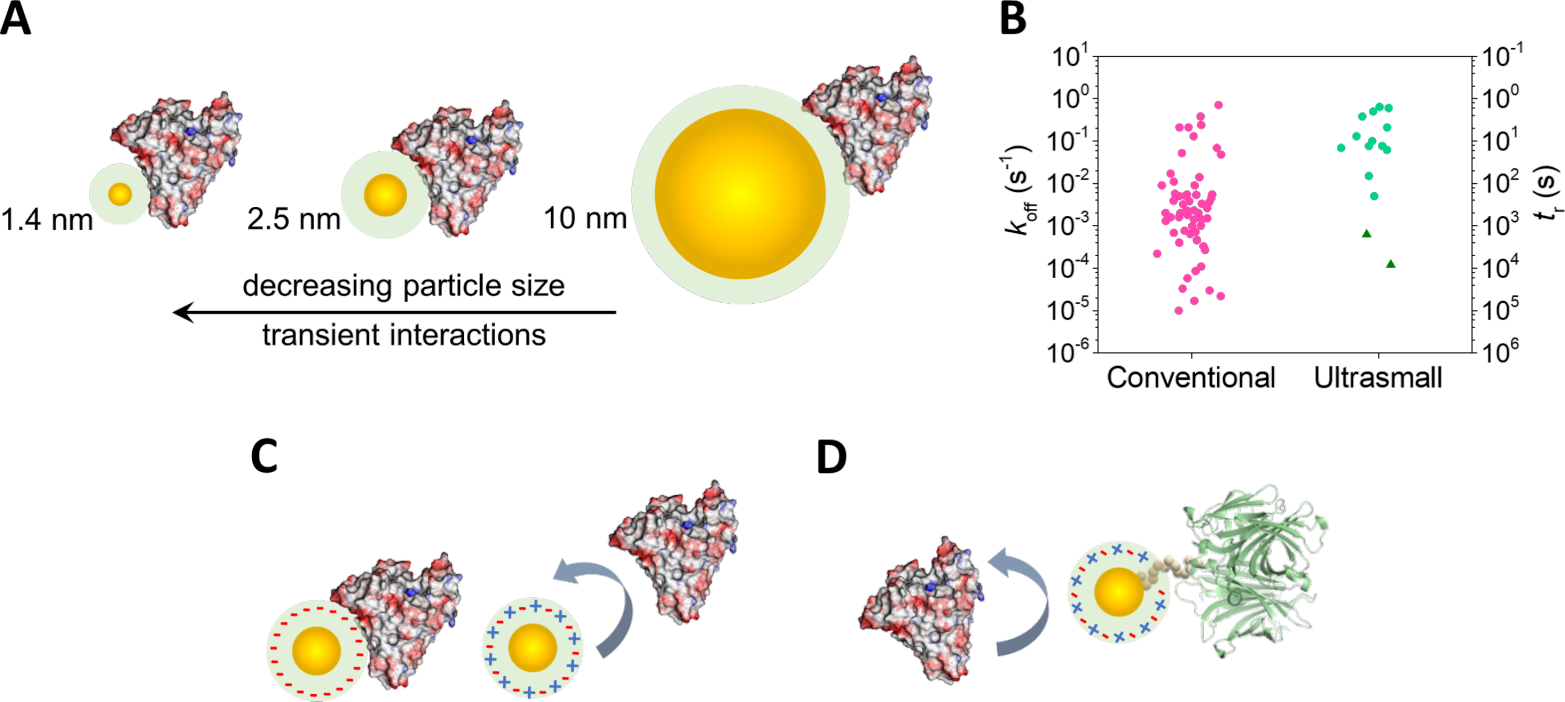
Ultrasmall NP–protein interactions. (A) Ultrasmall NPs form transient, short-lived complexes with proteins (albumin is shown as example; drawn to scale). (B) Compilation of apparent *k*_off_ and *t*_r_ values for NP–protein interactions. See [59] for additional information. (C) Ultrasmall NPs with proper surface chemistries (e.g., zwitterionic) can virtually eliminate nonspecific protein interactions. (D) Ultrasmall NPs can be functionalized to bind to target receptors without interference from nonspecific protein interactions. Figure 2A, 2C, and 2D were adapted from [58]. (“Biomolecular interactions of ultrasmall metallic nanoparticles and nanoclusters“, © 2021 Alioscka A. Sousa et al., published by the Royal Society of Chemistry, distributed under the terms of the Creative Commons Attribution Non-Commercial 3.0 Unported License, https://creativecommons.org/licenses/by-nc/3.0/). This content is not subject to CC-BY 4.0. Figure 2B was adapted from [59]. (© 2023 André F. Lima and Alioscka A. Sousa, published by MDPI, distributed under the terms of the Creative Commons Attribution 4.0 International License, https://creativecommons.org/licenses/by/4.0).

A distinguishing feature of usNPs is their transient, short-lived interactions with proteins (Figure 2A) [52–58]. This occurs because of the small size and high surface curvature of usNPs, which restrict the binding interface for proteins. As a result, protein spreading and denaturation on the usNP surface are minimized, and fewer non-covalent interactions form between usNPs and proteins compared to interactions between larger NPs and proteins. Quantitatively, Figure 2B compares experimentally determined apparent dissociation rate constants (*k*_off_) and corresponding residence times (*t*_r_ = 1/*k*_off_) for protein interactions with large (conventional) and ultrasmall NPs [59]. It can be discerned that residence times for non-targeted usNP–protein complexes range from a few seconds to a couple of minutes (Figure 2B, green circles), highlighting the short-lived nature of these interactions. In contrast, large NPs exhibit residence times that can extend to many hours, indicating the formation of a “permanently” bound (hard) protein corona. Moreover, given the appropriate combination of size and surface chemistry, nonspecific interactions between usNPs and proteins can be virtually eliminated (Figure 2C).

Notably, achieving highly stable and “stealth” usNPs is feasible through surface coating of the inorganic core with small molecules, such as glutathione (GSH), glucose, low molecular-weight polyethylene glycol (PEG), and various short peptides, among others [60–63]. This characteristic stands in sharp contrast to conventional large NPs, which often necessitate surface coating with bulkier molecules, such as long-chain PEG or various polymers. The strategic coating of usNPs with small molecules therefore preserves the overall ultrasmall size of the particles even within complex biofluids, such as human plasma. AuNCs coated with GSH exemplify this concept, displaying a small hydrodynamic diameter around 3 nm, outstanding colloidal stability, resistance to protein interactions, and absence of protein corona formation [61,64–65].

The efficient renal clearance of usNPs – typically >50% of the injected dose (ID) over 24 h – prevents their long-term accumulation in the organism [26–27,66]. Moreover, usNPs generally display significantly lower accumulation in the organs of the reticuloendothelial system (RES) compared to their larger counterparts [26,66]. For instance, certain AuNCs exhibit liver and spleen accumulation approximately 10–30 times lower than that of conventional NPs [66]. This reduced accumulation can be attributed to their efficient renal excretion together with the absence of stable interactions with blood proteins, especially those of the innate immune system that can mark NPs for phagocytosis by tissue-resident macrophages. Overall, usNPs are therefore more biocompatible than larger NPs. However, it is important to emphasize that the physicochemical and biological properties of usNPs are highly sensitive to NP size and surface chemistry [64,67–68], and usNPs can still impact protein activity, biochemical pathways, and cellular functions [54,69–75]. Therefore, a thorough evaluation of their biocompatibility is necessary before advancing their clinical applications.

In the context of cancer treatment, the efficient renal clearance and short blood elimination half-life of usNPs raise questions about their potential for tumor accumulation though passive targeting [76]. Fortunately, strategies to slow down renal clearance and extend the blood half-life of usNPs for more efficient tumor uptake are feasible, including fine-tuning hydrodynamic diameter (HD) through surface chemistry [77], controlling core density [78], and, potentially, modulating ultraweak nonspecific interactions with proteins [79]. For instance, Zheng and colleagues showed that AuNCs can be designed to demonstrate passive tumor targeting behavior comparable to that of larger NPs. GSH-coated AuNCs reached passive tumor uptake levels of 2–3% ID/g, while PEG-coated AuNCs displayed even higher passive tumor uptake efficiency of ≈8% ID/g owing to their longer blood retention time [77]. Besides achieving decent tumor uptake levels in some cases, usNPs exhibit easier penetration and diffusion through the dense tumor microenvironment relative to their larger counterparts [80–82].

However, a potential downside of usNPs resides in their more rapid efflux from the tumor tissue. This challenge could be potentially addressed through the utilization of actively targeted particles. Importantly, usNPs functionalized with targeting ligands exhibit behavior akin to bioactive proteins, facilitating interactions with cognate cell-surface receptors with reduced interference, if any, from the formation of an adsorbed protein corona (Figure 2D). Actively targeted usNPs are discussed in further detail below.

### 3 Actively targeted ultrasmall NPs

The incorporation of active targeting strategies is expected to further enhance the selectivity and performance of usNPs for cancer treatment. By designing usNPs to target surface receptors on cancer cells, tumor retention can be improved by minimizing particle intravasation back to tumor blood vessels. Active targeting can also promote usNP transport to the cell interior, potentially leading to more effective drug delivery and chemotherapy. It must be noted that the success of these strategies relies on efficient passive targeting in the first place [83–84]. Nevertheless, cumulative evidence suggests that actively targeted usNPs can enhance tumor accumulation compared to non-targeted particles (Section 5). Furthermore, usNPs containing tumor homing and penetrating peptides can target the more accessible tumor vasculature, potentially aiding in particle accumulation within the tumor site [85–86].

A direct comparison of the impact of NP size on the tumor accumulation and retention of actively targeted particles was undertaken by Xu and colleagues [87]. The authors synthesized transferrin-coated iron oxide NPs with core sizes of 3 and 30 nm and assessed their binding to transferrin receptors overexpressed in a 4T1 xenograft breast cancer model. Their findings revealed that actively targeted 3 nm NPs produced a sixfold higher level of tumor retention compared to non-targeted counterparts. In contrast, the corresponding improvement in tumor retention was only 1.15-fold in the case of the larger NPs. This difference was attributed to easier tumor clearance (tumor intravasation back to blood vessels) of off-targeted 3 nm NPs compared to 30 nm ones.

Common functional ligands employed in actively targeted usNPs encompass small molecules such as folate, aptamers, peptides, full antibodies, and antibody fragments. These ligands can be covalently attached to the underlying surface coat through standard bioconjugation chemistry or utilizing bioorthogonal bioconjugation strategies such as click-chemistry [88–89]. Additionally, in certain situations, the targeting ligand can be directly conjugated to the NP inorganic core, exemplified by the S–Au bond formed between cysteine-containing molecules and gold NPs [90–92]. Actively targeted AuNCs can also be prepared using bioactive peptides or proteins via a one-step biomineralization process, in which case the peptide or protein serves the purpose of both surface stabilization and functionalization [93–95]. Importantly, the use of antibodies and other proteins as targeting agents may increase the HD of usNPs beyond the threshold for renal filtration, and so careful consideration is needed in the design of such constructs.

To ensure effective interaction with cell surface receptors on cancer cells, the incorporation of targeting ligands onto usNPs must optimize the exposure, orientation, and conformation of the functional portion. For small molecules and peptides in particular, the functional moiety must circumvent both steric hindrance from the underlying surface coat and undesired intermolecular interactions on the ligand shell. In this regard, computer simulations emerge as a powerful tool for optimizing the size and composition of usNPs designed for receptor targeting. For example, Häkkinen and colleagues designed a series of 1.7 nm AuNCs functionalized with RGD peptides as targeting ligands along with chemotherapy drugs and inhibitors of signaling pathways [96–97]. Their simulations revealed that the system composition and the peptide/drug ratio critically influenced the targeting ability of the particles. In addition to computer simulations, a detailed experimental characterization of the surface properties and interactions of targeted usNPs is indispensable for elucidating their biological behavior and optimizing their performance.

### 4 The significance of binding affinity and kinetics

It is imperative to assess the apparent binding affinity (*K*_D_, *K*_i_, or IC_50_) between targeted usNPs and their target receptors. Despite the known *K*_D_ for the free ligand binding to the receptor (Table 1) [98–112], the effective *K*_D_ may differ when the same ligand is attached to a NP surface, possibly because of conformational changes or intermolecular interactions within the capping layer. Another intriguing aspect is understanding how the apparent binding affinity varies with the number of attached ligands. On one hand, attaching multiple ligands on a single usNP may enhance binding affinity through avidity effects. On the other hand, too many ligands could alter the original surface characteristics of usNPs, leading to stronger nonspecific interactions with plasma proteins.

**Table 1:** Receptor/ligand combinations employed in active targeting strategies involving usNPs for cancer diagnosis and treatment.

Ligand	Receptor	Receptor expression	Binding affinity^a^	Ref.

folic acid	folate receptor	Receptor is overexpressed in various cancers. Presents low to negligible expression in normal tissues.	<1 nM	[30,89,98,113–117]
RGD motif^b^	αvβ3 and αvβ5 integrin receptors	Receptors are overexpressed on angiogenic blood vessels and tumor cells, while being essentially absent in normal vessels.	1–100 nM	[32,51,100,118–124]
CendR motif (e.g., CRGDK)	neuropilin-1 (NRP1) receptor	Receptor is overexpressed in various cancers.	1.4 μM	[101,125]
α-melanocyte-stimulating hormone (αMSH) peptide analogs	melanocortin-1 receptor	Receptor is overexpressed on human melanoma tumor cells.	0.2–6 nM	[106–107,126–128]
PSMA-1 peptide-based^c^	PSMA receptor	Receptor is highly expressed on prostate cancer cells.	2 nM	[102–103,111,129–131]
bombesin peptide	gastrin releasing peptide (GRP) receptor	Receptor is frequently expressed on various cancers, including colorectal, pancreas, prostate, and breast.	4 nM	[104,132]
luteinizing hormone- releasing hormone (LHRH)	LHRH receptor	Receptor is overexpressed in the majority of cancers. Apart from pituitary cells, its expression in healthy tissues is limited.	5 nM	[105,133]
extracellular loop 1 inverso peptide (ECL1i)	chemokine receptors (CCR2)	The CCL2/CCR2 axis is involved in inflammatory responses and the growth and metastasis of many tumors, including breast carcinoma and pancreatic ductal adenocarcinoma.	2 μM	[112,134–135]
cyclic peptides MCP and FC131; small molecule plerixafor	chemokine receptors (CCR4)	CXCR4 is reported to be overexpressed in glioblastoma and in breast cancer primary tumors. It is also critical for invasion and metastases.	5 nM; 20 nM; 600 nM	[136–141]
AS1411, DNA aptamer	nucleolin (NCL) receptor	Receptor is selectively expressed on the surface of tumor cells. It is also found in the intracellular space of normal cells.	169 nM	[108,142–144]
Anti-HER2 antibody	HER2 receptor	Receptor is overexpressed in 15–30% of breast cancers. Overexpression also occurs in other malignancies like ovarian, stomach, and lung adenocarcinoma.	10 nM	[109,145–146]
Anti-CD326 antibody^d^	epithelial cell adhesion molecule (CD326) receptor	CD326 is overexpressed in the majority of cancer tissues.	1 nM–2 μM	[110,147]
Anti-BCMA antibody	BCMA	BCMA is preferentially expressed by mature B lymphocytes.	<1 nM	[148–150]

^a^Apparent binding affinities estimated for the free ligand binding to corresponding receptor. The binding affinity could differ when the ligand is immobilized on an usNP. Some entries report direct *K*_D_ measurements, while others report IC_50_ or *K*_i_ values determined from competition assays. ^b^Kapp et al. performed a comprehensive evaluation of the binding affinity (IC_50_ values) of different RGD peptide ligands to various integrin receptors [100]. While short linear peptides demonstrated binding to the αvβ3 integrin with affinities ranging from 12 to 89 nM, short cyclic peptides displayed stronger affinities in the range of 1.5 to 6 nM. ^c^Basilion and colleagues developed a peptide-based high-affinity ligand for PMSA, referred to as PSMA-1 [103]. Moreover, a recent review highlights the latest developments in PSMA-targeted therapy for prostate cancer [111]. ^d^Affinity values for five distinct antibodies were reported by Münz et al. [110].

Targeted usNPs with weak binding to cancer cell surface receptors may not provide any additional value over non-targeted particles. As stressed by Ruoslahti and colleagues [86,151], many peptide ligands bind their receptors with weak affinities in the high-nanomolar to low-micromolar range. This implies that delivering a substantial excess of targeted usNPs locally would be needed for receptor saturation ([usNP] = 9 × *K*_D_ for 90% saturation), but challenges with insufficient NP tumor penetration and diffusion make this unlikely. Even if delivering a high local concentration of targeted usNPs were possible, the contribution of active targeting would not be distinguishable from the nonspecific background in this case. To address the challenge of weak ligand–receptor affinity, one can opt for a more suitable high-affinity ligand, or design usNPs to leverage avidity effects.

Another layer of complexity arises from the in vivo system operating in an open, non-equilibrium state, where concentrations constantly change and biological processes are dynamically regulated [152–153]. Consequently, it becomes important to extend the characterization beyond binding affinity and include the examination of binding kinetics between targeted usNPs and their receptors [59]. For a simple one-step binding model, *K*_D_ = *k*_off_/*k*_on_ and *t*_r_ = 1/*k*_off_, where *k*_on_ and *k*_off_ are the association and dissociation rate constants of the binding reaction, respectively, and *t*_r_ is the residence time of the complex. The value of *k*_off_ (or *t*_r_) is determined by short-range non-covalent interactions at the binding interface, reflecting the stability of the bound complex. For instance, with a *K*_D_ of 1 nM and a characteristic *k*_on_ of 1 × 10^6^ M^−1^·s^−1^, the residence time would be approximately 17 min. In chemically related compounds, *k*_on_ generally remains more or less invariant, and relative changes in *K*_D_ follow corresponding changes in *k*_off_ [152–153]. Avidity effects also manifest through a reduction in *k*_off_ while *k*_on_ remains unaffected. Importantly, a prolonged residence time may prove beneficial when targeting the tumor vasculature, as the targeted usNPs would remain bound to their receptors even as most of the circulating particles are cleared from the body. Furthermore, a prolonged residence time could be advantageous for retaining usNPs within the tumor, particularly for the smallest particles (e.g., few-atom AuNCs) that may experience not only efficient renal clearance but also rapid efflux from the tumor. It is noteworthy that this concept has been experimentally demonstrated in a mouse xenograft model using very small DARPin proteins (14.5 kDa) with a range of affinities (0.09 to 270 nM through differences in *k*_off_) for the HER2 receptor [154]. It was found that the highest-affinity DARPin reached 8% ID/g tumor accumulation, whereas the lowest-affinity DARPin reached only 0.6% ID/g. These results were consistent with a modeling analysis of the effects of molecular size and binding affinity on tumor accumulation, developed by Schmidt and Wittrup [155].

### 5 Pre-clinical applications of targeted ultrasmall NPs

In this section, we present a selection of pre-clinical applications involving targeted usNPs, with a focus on animal studies over in vitro investigations. We highlight twelve studies that explore seven distinct ligand–receptor combinations (see subsections 5.1 through 5.7). Our aim was to review a diverse range of ligand–receptor pairs, covering small molecule-, peptide-, aptamer-, and antibody-based ligands used in active targeting. While not exhaustive, we hope the highlighted cases below provide a valuable overview of the capabilities and the potential of targeted usNPs in cancer nanomedicine. Furthermore, Table 1 presents a compilation of 13 unique ligand–receptor combinations employed in active targeting of usNPs, featured in over 45 published reports [30,32,51,89,99,106–107,113–138,142–147,149–150,156–159]; and Table 2 provides a quantitative assessment of tumor uptake for actively targeted usNPs compared to control groups, limited to studies including quantitative values of % ID/g.

**Table 2:** Quantitative assessment of tumor uptake for actively targeted usNPs compared to control groups.

NPs^a^	Targeting ligand	Cancer type / Tumor model	Uptake (ID/g)	Uptake (ID/g)	Time p.i. (h)	Designation of control groups	Ref.

			Targeted	Control			

CuNCs	LHRH peptide	lung cancer A549 (sc)	12%	3%	4	unconjugated CuNCs	[133]
CuNCs	LHRH peptide	lung cancer A549 (orthotopic)	10%	5.2%	4	unconjugated CuNCs	[133]
usAuNPs	TAT peptide	liver cancer LM3	9.6%	2.7%	24	non-cleavable linker between TAT & NPs	[156]
AuNCs^b^	AS1411 aptamer	breast cancer 4T1	7.8%	4.2%	24	unconjugated AuNCs	[143]
AuNCs^c^	c(RGDyC) peptide	breast cancer 4T1	6.4%	2%	4	AuNCs coated with control c(RADyC)	[122]
AuNCs^c^	Anti-CD326 antibody	breast cancer MCF-7	12%	3%	24	unconjugated AuNCs	[147]
AuNCs	Glucose	breast cancer MDA-MB-231	3.4%	1.3%	12	glutathione-coated AuNCs	[158]
AuNCs	PSMA-binding peptide	prostate cancer PC3pip	8.9%	2%	4	unconjugated AuNCs in PSMA-negative tumors	[129]
Cu-AuNCs^c^	ECL1i	breast cancer 4T1	19.4%	5.6%	1	unconjugated Cu-AuNCs	[135]
Cu-AuNCs	FC131	glioblastoma multiforme / U87	4.6%	1.1%	24	unconjugated Cu-AuNCs	[137]
Mn-Iron oxide NP	MCP	breast cancer MCF-7	9.8%	4.1%	1	unconjugated NPs	[136]
AGuIX	Anti-BMCA antibody	multiple myeloma	4.4%^d^	0.1%	0.5	unconjugated NPs	[149]
AGuIX	Anti-BMCA antibody	multiple myeloma	8.2%^d^	2.1%	<2	unconjugated NPs	[150]
AGuIX	Anti-MUC1-C antibody	breast cancer E0771	6.6%	5.9%	1	unconjugated NPs	[159]
C' dots	Anti-HER2 scFv	gastric cancer NCI-N87	10.7%	5.7%	72	NPs with scFv isotype control	[146]
C' dots	αMSH peptide	melanoma B16F10	5%	2.5%	48	inhibitor co-injected in excess	[107]
C' dots	cRGDY peptide	melanoma M21	3%	1%	24	αvβ3-negative tumors	[119]
C' dots	cRGDY peptide	melanoma M21	12%	3%	24	αvβ3-negative tumors	[120]
C' dots	Anti-HER2 scFv	breast cancer BT-474	13.2%	5%	24	scFv isotype control; HER2-negative tumors	[145]
C' dots	αMSH peptide	melanoma M21	9.3%	4.6%	24	peptide agonist co-injected in excess	[128]
C' dots	PSMA-binding peptide	prostate cancer LNCap	8.1%	3.9%	12	PSMA-negative PC-3 tumors	[130]

^a^CuNCs, copper nanoclusters; usAuNPs, ultrasmall gold NPs; AuNCs, gold nanoclusters; AGuIX, Gd-chelated polysiloxane NPs; C’ dots, Cornell dots (core–shell silica NPs), ^b^% ID/g was determined from the information provided in the manuscript, ^c^Tumor uptake reported as % ID, ^d^Uptake detected in the spine.

#### 5.1 Folic acid ligand/folate receptor

**Figure 3:**
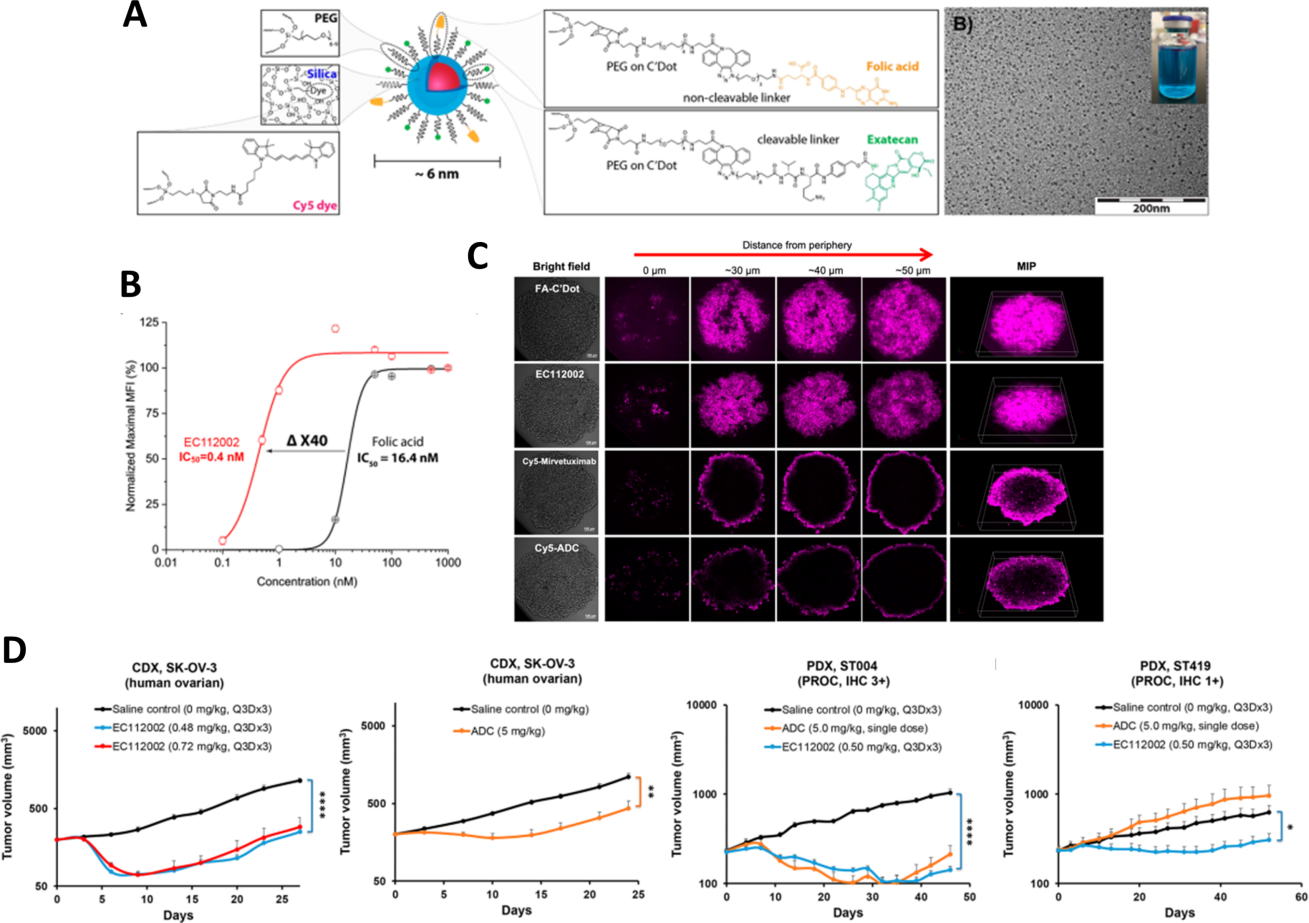
Folic acid-targeted and drug-conjugated C’ dots for enhanced tumor penetration and therapeutic efficacy. (A) Schematic illustration outlining the nanoscale architecture of multifunctional C’ dots. (B) Competitive cell-binding assay of targeted usNPs (EC112002) revealed a 40-fold improvement in affinity for folate receptor binding compared to free folic acid. (C) Z-stacks of confocal microscopy images of a tumor spheroid treated with targeted usNPs (first and second rows) and antibodies (third and fourth rows), with and without the conjugated drug. The usNPs demonstrated deeper tumor penetration and more uniform distribution compared to the antibody. (D) In vivo efficacy of targeted usNPs in SK-OV-3 human ovarian cancer bearing mice and in patient-derived xenografts (PDX). The usNPs showed superior therapeutic efficacy compared to an antibody drug conjugate (ADC). The figure was adapted with permission from [115]. Copyright 2022 American Chemical Society. This content is not subject to CC BY 4.0.

Wu et al. developed core–shell silica NPs (Cornell dots, or C’ dots) composed of encapsulated Cy5 near-infrared (NIR) dyes, a protective PEG layer, the drug exatecan conjugated via a cathepsin-B cleavable linker, and folic acid targeting molecules (Figure 3A) [115]. The multifunctional C’ dot particles contained on average 21 exatecan and 13 folic acid molecules, while maintaining a compact HD of about 6.4 nm. As a result of their ultrasmall size and protein-resistant surface chemistry, the C’ dots showed efficient renal clearance and no significant retention in any critical organ. A competitive cell-binding study was conducted to assess the binding affinity of the targeted C’ dots toward corresponding folic acid receptors (Figure 3B). The findings revealed a strong multivalency effect, leading to a 40-fold enhancement in binding affinity relative to free folic acid (IC_50_ values of 0.4 nM vs 16.4 nM). Systematic studies were conducted to compare the performance of the targeted C’ dot formulation with an antibody drug conjugate using both 3D tumor spheroid models and xenograft animal tumor models. The results indicated that the C’ dots exhibited significantly deeper penetration within 3D cell-line-derived spheroids (Figure 3C). Additionally, the particles demonstrated enhanced efficacy in both cell-line-derived and patient-derived in vivo tumor xenograft models (Figure 3D).

#### 5.2 RGD peptide ligands/integrin receptors

**Figure 4:**
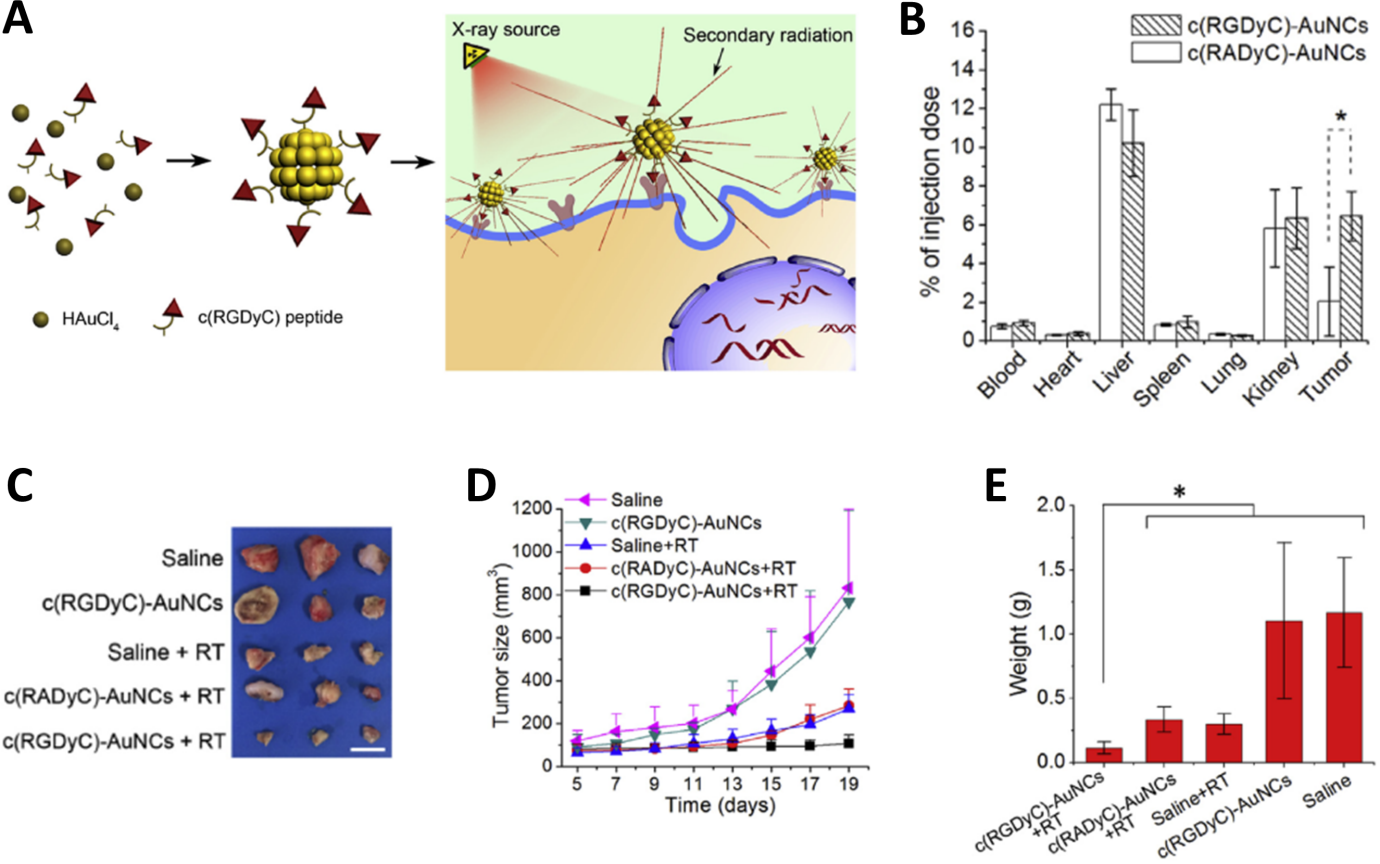
RGD peptide-functionalized AuNCs as tumor-targeted radiotherapy sensitizers. (A) Preparation of AuNCs through a one-pot synthesis with c(RGDyc) peptides. The targeted AuNCs were evaluated as radiotherapy sensitizers in tumor-bearing mice. (B) Biodistribution, including tumor accumulation, of targeted vs non-targeted AuNCs. (C) Photographs of dissected tumor tissues following treatment. (D) Tumor volume growth curves and (E) ex vivo weight of tumors at 14 days after treatment. Treatments included saline (control), targeted AuNCs, non-targeted AuNCs, with or without radiotherapy (RT). The figure was adapted from [122], Biomaterials, vol. 144, by G. Liang; X. Jin; S. Zhang; D. Xing, “RGD peptide-modified fluorescent gold nanoclusters as highly efficient tumor-targeted radiotherapy sensitizers”, pages 95–104, Copyright (2017), with permission from Elsevier. This content is not subject to CC BY 4.0.

Liang et al. prepared luminescent AuNCs coated with c(RGDyC) in a one-pot synthesis (Figure 4A) [122]. The peptide comprised two functional parts: one part (Tyr and Cys residues) was responsible for reducing Au^3+^ into AuNCs, while the other part (RGD sequence) targeted αvβ3 integrin receptors. In murine models, the AuNCs were non-toxic and underwent renal clearance. Upon intravenous administration to 4T1 breast cancer tumor-bearing mice, the AuNCs reached a tumor accumulation of 6.4% ID at 4 h p.i, significantly higher than the 2% ID achieved with AuNCs coated with the non-targeting control peptide c(RADyC) (Figure 4B). The targeted AuNCs accumulated in the tumor region acted as radiosensitizers, markedly enhancing the efficacy of radiotherapy and delaying tumor growth (Figure 4C–E).

Chen et al. designed dye-labeled (Cy5) and radioiodinated (^124^I or ^131^I) C’ dots for dual-modality NIR optical and positron emission tomography (PET) tumor imaging. Furthermore, the particles contained the cRGDY peptide for the targeting of αvβ3-positive human melanoma (M21) tumor-bearing mice [119]. The authors tested different cRGDY ligand numbers (6, 14 or 18) to understand how variations in ligand density impacted essential biological activities such as clearance, pharmacokinetics, and targeted tumor accumulation in M21 xenografts. It was observed that all C’ dots underwent efficient renal clearance, with over 90% ID excreted over 3 days p.i. At 4 days p.i., the targeted C’ dots were nearly entirely eliminated from the carcass, especially the 18-ligand functionalized particles, which showed over 98% elimination. Moreover, there was no significant uptake observed in the RES or other major organs. Although variations in ligand number did not affect the magnitude of accumulated radioiodine activity in tumors, tumor-to-blood ratios reached a peak value of ≈10 for the C’ dots with 18 ligands. To further validate tumor-specific targeting, the 18-ligand C’ dots were administered to both M21 and M21-L (αvβ3-negative) tumor-bearing mice, and imaging was performed using a PET system. The results showed increased uptake of targeted C’ dots in M21 compared to M21-L xenografts from 4 to 96 h p.i., with the maximum difference at 24 h p.i. being ≈3 vs 1% ID/g.

#### 5.3 Melanocyte-stimulating hormone peptide/melanocortin-1 receptor

Zhang et al. developed actively targeted C’ dots for improving radiotherapy in melanoma-bearing mice [128]. The C’ dots were designed to display around 12 α-melanocyte-stimulating hormone (αMSH) peptide analogs for targeting the melanocortin-1 receptor (MC1-R) overexpressed on human melanoma cells (M21). In addition, the NPs were radiolabeled with ^177^Lu and fluorescently labeled with Cy5. A competitive cell-binding assay demonstrated that, due to avidity effects, the targeted C’ dots exhibited a ≈10-fold improvement in binding affinity to MC1-R relative to the free peptide, with IC_50_ values of 0.41 nM vs 3.3 nM. The particles had an HD of ≈6 nm and were readily cleared through the urine, with 40% and 80% ID excreted at 24 and 96 h p.i., respectively. In M21 xenografts, the targeted C’ dots reached tumor accumulations of 5.5 and 9.3% ID/g at 4 and 24 h p.i., respectively. The importance of active targeting was demonstrated by co-injecting an excess of a high-affinity peptide agonist, resulting in approximately 50% blocking of tumor uptake. Tumor-bearing mice treated with targeted and radiolabeled C’ dots showed prolonged survival relative to control groups. Animals treated with 0.5 mCi (milliCurie) of targeted C’ dots demonstrated improved survival compared to those treated with the same dose of non-targeting C’ dots.

#### 5.4 PSMA-targeting ligands/PMSA receptor

**Figure 5:**
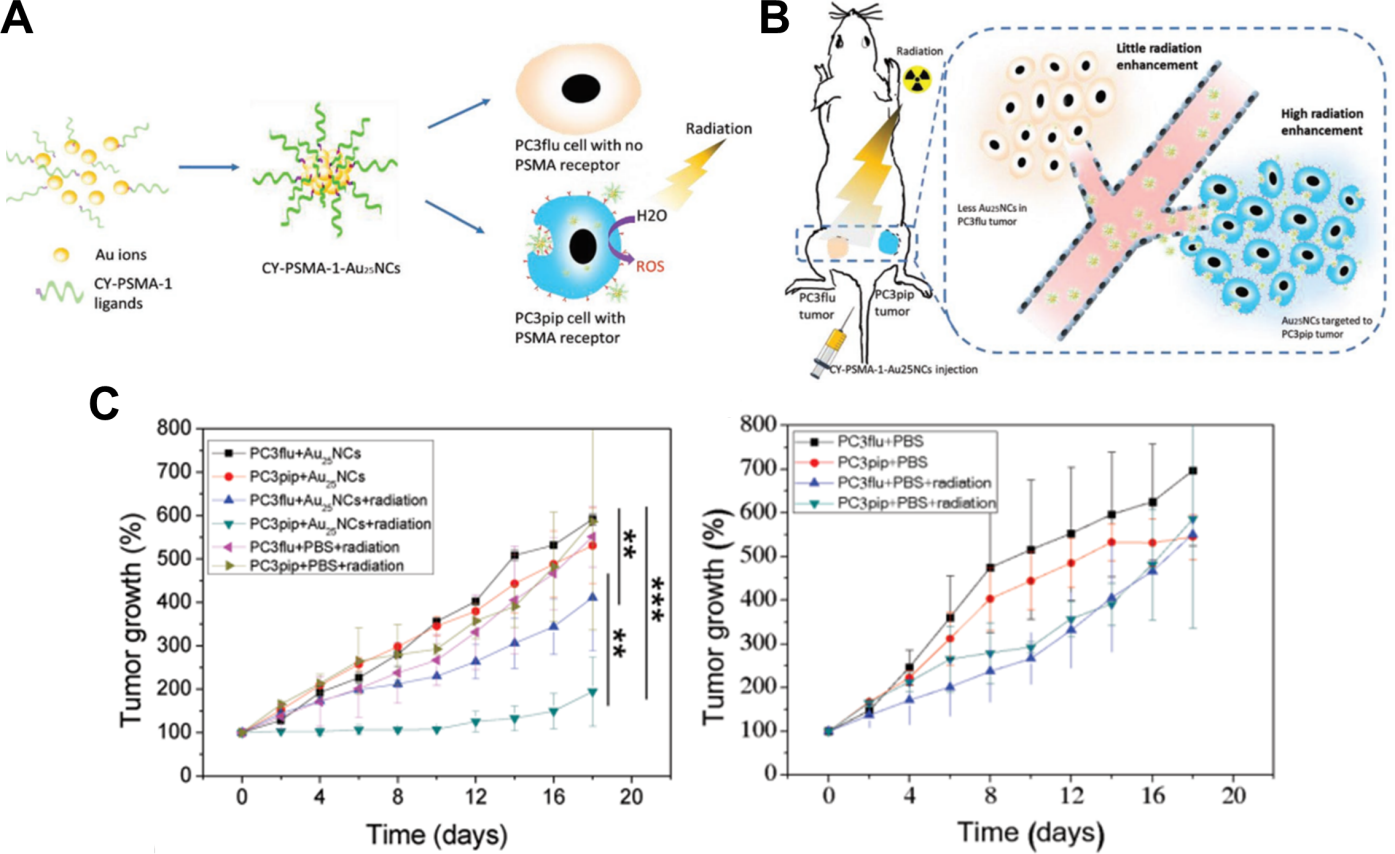
PSMA-1-targeted AuNCs for enhanced radiotherapy of prostate cancer. (A) Schematic illustration of targeted AuNCs and their binding selectivity to PSMA-positive (PC3pip) over PSMA-negative (PC3flu) prostate cancer cell. (B) Illustration of intravenous injection of AuNCs, highlighting improved targeting levels and enhanced radiation therapy in PSMA-expressing tumors. (C) Tumor growth curves for PC3pip and PC3flu tumor-bearing mice treated with targeted AuNCs or PBS, with or without radiation. Significant reduction in tumor growth was observed in the PC3pip model 18 days after treatment with targeted AuNCs plus radiation. The figure was adapted from [129], D. Luo et al., “Targeted Gold Nanocluster Enhanced Radiotherapy of Prostate Cancer”, Small, with permission from John Wiley and Sons. Copyright © 2019 WILEY-VCH Verlag GmbH & Co. KGaA, Weinheim. Germany. This content is not subject to CC BY 4.0.

Luo et al. developed AuNCs covered with PSMA-1 for selective prostate cancer targeting and radiotherapy enhancement (Figure 5A,B) [129]. In this design, the PSMA-1 ligand was modified to contain additional Cys and Tyr residues to promote nanocluster formation through a biomineralization process. Binding of the targeted AuNCs to PSMA-expressing cells was evaluated using a competition binding assay. Compared to free PSMA-1 ligands, the targeted AuNCs demonstrated significantly reduced binding affinity (IC_50_ values of 1.7 nM vs 0.09 nM), likely attributed to avidity effects. Importantly, the particles maintained a small HD of ≈3 nm, supporting their efficient renal clearance. In vivo experiments performed in tumor-bearing mice revealed that the Au content in PSMA-expressing tumors was two- to threefold higher than in tumors lacking PSMA expression. Furthermore, the targeted AuNCs reached a tumor accumulation of around 8.9% ID/g compared to 2% ID/g for non-targeted particles at 4 h p.i., highlighting the superior efficacy of the active targeting strategy over EPR-based passive targeting. X-ray irradiation of tumor-bearing mice treated with targeted AuNCs demonstrated a significant enhancement in radiotherapy effectiveness (Figure 5C). In combination, the selective targeting and rapid clearance of PSMA-targeting AuNCs offers the potential for reducing radiation dosage and minimizing exposure to healthy tissue.

Chen et al. developed renal-clearable and PSMA-targeting C’ dots for dual-modality imaging of prostate cancer [130]. These particles featured a ≈4 nm silica core and multiple functionalities, including encapsulated Cy5 NIR dyes, ^89^Zr radiolabels, and peptide-based PSMA-targeting ligands. Competitive cell-binding assays using a high-PSMA-expressing cell line (LNCap) revealed that the targeted C’ dots exhibited at least a twofold improvement in the IC_50_ value (1.8 nM) compared to the free PMSA-targeting ligand (4.5 nM). Urinary clearance in healthy mice was found to be 26% ID at 4 h p.i., reaching a total clearance of 53% ID at day 7. Biodistribution studies performed 24 h p.i. revealed that the particles accumulated to 5% ID/g or less in major organs, including the liver, kidney, and salivary glands. In tumor-bearing mice, microPET imaging revealed over a twofold higher tumor uptake in LNCap mice (8.1% ID/g) compared to control PC-3 mice (3.9% ID/g) at 24 hours p.i., with peak tumor uptake in LNCap mice occurring at 48 h p.i. (≈9% ID/g).

#### 5.5 AS1411 aptamer ligand/nucleolin receptor

Chen et al. prepared AuNCs (≈3 nm) stabilized with histidine amino acids [142]. These particles were then functionalized through chemical conjugation with two targeting ligands, cyclic RGD (cRGD) and AS1411, along with the incorporation of doxorubicin (DOX) or the near-infrared (NIR) dye MPA. Tumor-bearing mice were employed to assess the tumor specificity of AuNC-MPA, AuNC-MPA-cRGD, and AuNC-MPA-cRGD-AS1411 using in vivo NIR fluorescence imaging. Both targeted AuNCs accumulated in the tumor, while the control AuNC-MPA showed negligible accumulation. However, the contrast ratio of tumor to normal tissue was higher for AuNC-MPA-cRGD-AS1411, reaching a value of 7.2 compared to 5.4 for AuNC-MPA-cRGD. This finding indicates that the dual-targeted particles had superior tumor-targeting ability. Furthermore, enhanced tumor therapy was demonstrated in a mouse tumor model with AuNC-DOX-cRGD-AS1411.

#### 5.6 Chemokine receptor ligands/chemokine receptors

**Figure 6:**
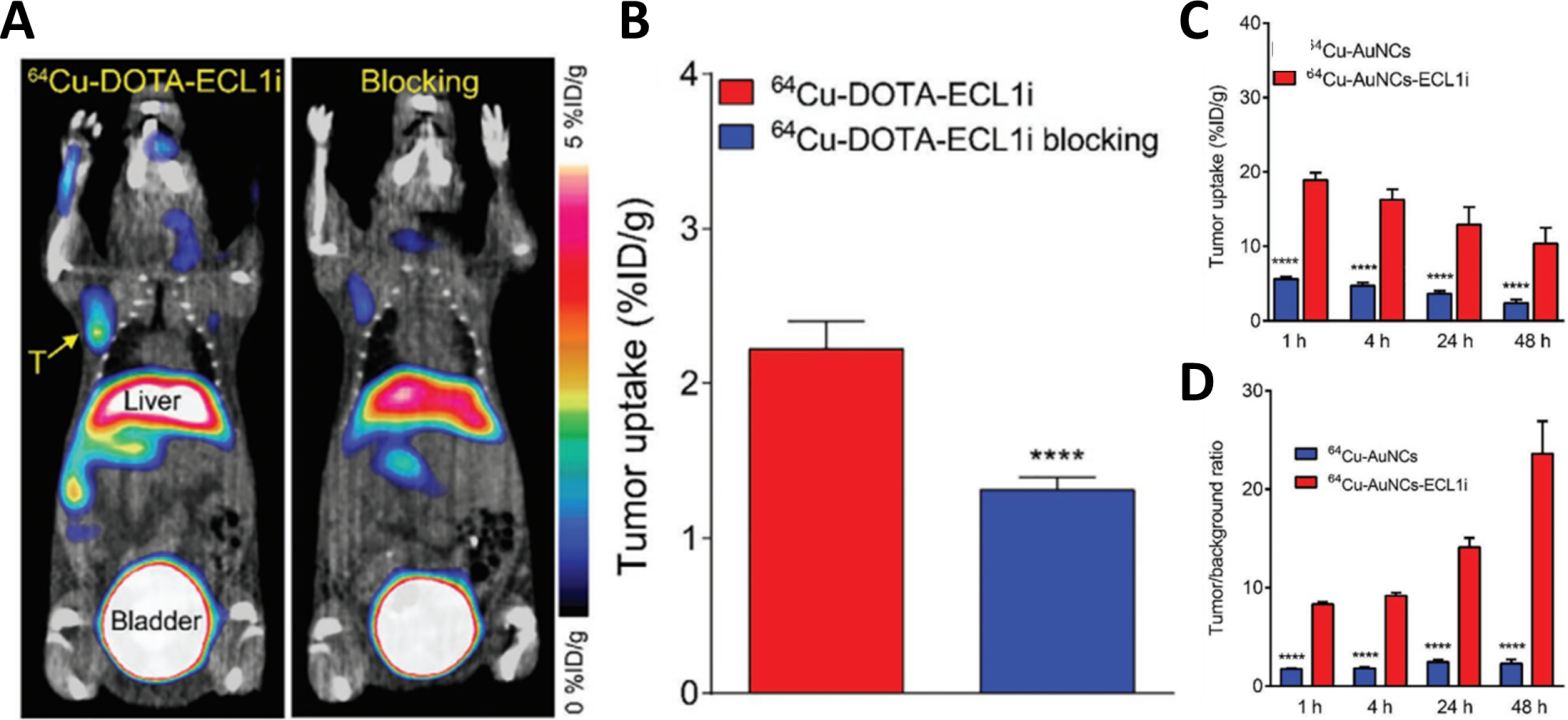
PET images detecting CCR2 in a mouse TNBC model using ^64^Cu-AuNCs-ECL1i. (A, B) Assessment of CCR2 targeting specificity using a peptide radiotracer ^64^Cu-DOTA-ECL1i and a non-radiolabeled ECL1i peptide for blocking. (C) Tumor uptake and (D) tumor/background ratio of CCR2-targeted ^64^Cu-AuNCs-ECL1i compared to non-targeted ^64^Cu-AuNCs along a 48 h period. The figure was adapted from [135], Y. Zhao et al., “Chemokine Receptor 2 Targeted Gold Nanocluster Imaging Triple Negative Breast Cancer with Positron Emission Tomography”, Particle & Particle Systems Characterization, with permission from John Wiley and Sons. Copyright © 2021 WILEY-VCH Verlag GmbH & Co. KGaA, Weinheim. Germany. This content is not subject to CC BY 4.0.

Recently, Zhao et al. reported the development of a chemokine receptor 2 (CCR2)-targeted and renal clearable radiolabeled gold nanocluster, ^64^Cu-AuNCs-ECL1i, for triple negative breast cancer (TNBC) PET imaging [135] (Figure 6). ^64^Cu-AuNCs-ECL1i had a uniform core size of 2.5 nm, hydrodynamic diameter of 5.1 nm, and zeta potential of 6.8 ± 1.6 mV. Notably, the ECL1i peptide (DLeu-Gly-DThr-DPhe-DLeu-DLys-DCys) was previously shown to selectively inhibit CCL2-induced chemotaxis (IC_50_ = 2 µM) [112]. In fact, the CCL2/CCR2 signaling axis plays a crucial role in cancer development by facilitating the proliferation and invasion of tumor cells and recruiting immunosuppressive cells, thus representing a significant opportunity for targeted therapy [160]. Results from a mouse model of TNBC showed improved tumor uptake of ^64^Cu-AuNCs-ECL1i compared to non-targeted ^64^Cu-AuNCs (19.4 vs 5.6% ID/g at 1 h p.i.) (Figure 6C). Moreover, the results revealed a notable increase in tumor/background contrast ratio over the course of 48 h for the targeted vs the non-targeted particles (Figure 6D).

Zhang et al. have also engineered ultrasmall and renal clearable ^64^Cu-radiolabeled nanoparticles with ECL1i [134]. These particles were employed not only for targeted PET imaging but also for loading and delivery of gemcitabine (^64^Cu-Cu@CuO_x_-ECL1i-Gem) into pancreatic ductal adenocarcinoma (PDAC). In this study, an innovative strategy was used to prepare 5.3 nm diameter ^64^Cu-Cu@CuO_x_-ECL1i NPs and to covalently conjugate gemcitabine, improving drug stability and prolonging the circulation half-life. Moreover, after accumulation in the tumoral regions due to CCR2 targeting, the drug could be released within the acidic tumor microenvironment, thus enhancing treatment efficacy. The study revealed that ^64^Cu-Cu@CuO_x_-ECL1i exhibited suitable biodistribution and biocompatibility. Moreover, ^64^Cu-Cu@CuO_x_-ECL1i-Gem was able to induce tumor inhibition and to prolong survival in a syngeneic xenograft mouse model of PDAC.

#### 5.7 Antibody-based ligands/receptors

**Figure 7:**
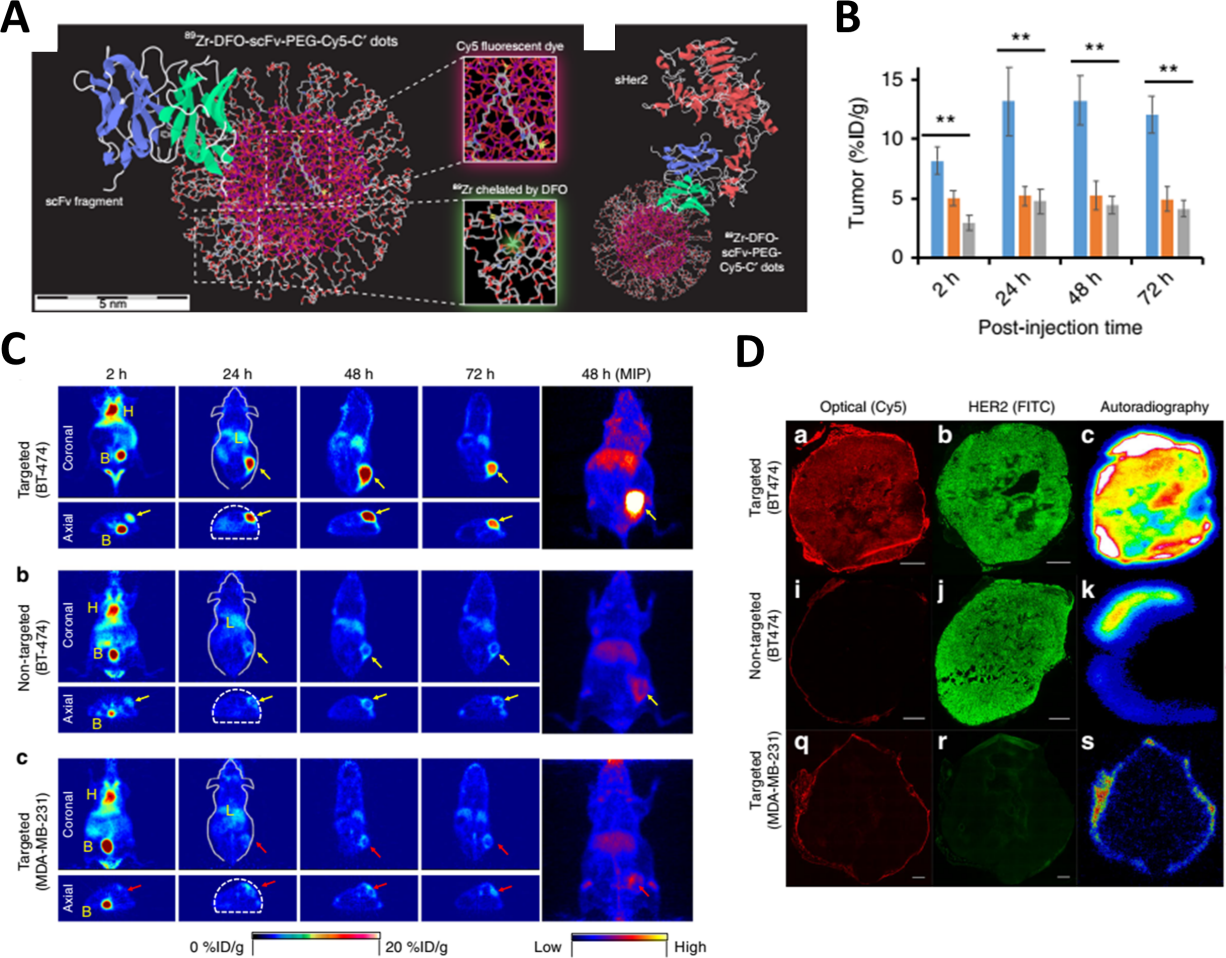
Radiolabeled and anti-HER2-targeted C’ dots for diagnosis and treatment of HER-2 overexpressing breast cancer. (A) Schematic illustration outlining the nanoscale architecture of multifunctional C’ dots. (B, C) Tumor uptake comparison between targeted and non-targeted C’ dots (blue and orange bars in B, respectively) in HER2-positive BT-474 tumors, along with the uptake of targeted C’ dots (gray bars in B) in HER2-negative MDA-MB-231 tumors. Corresponding PET images are depicted in C. MIP refers to maximum intensity projections, acquired at 48 h p.i. (D) Ex vivo tumor histopathology featuring Cy5-fluorescence microscopy, HER2 immunohistochemical staining, and autoradiography images. This figure was adapted from [145] (© 2018 Feng Chen et al., published by Springer Nature, distributed under the terms of the Creative Commons Attribution 4.0 International License, https://creativecommons.org/licenses/by/4.0).

Chen et al. developed multifunctional C’ dot particles integrating encapsulated Cy5 dyes, stealth PEG layer, ^89^Zr radiolabels, and 25-kDa anti-HER2 scFv fragments (Figure 7A) [145]. The surface density of each functional group was precisely controlled to yield a final construct of 7.3 nm in size. As a result of their small size and stealth nature, the C’ dots exhibited efficient renal clearance, low nonspecific RES accumulation, and an estimated whole-body excretion value of 70% ID over 72 h p.i. Competitive cell-binding assays with HER2-expressing BT-474 cells revealed similar HER2 receptor binding affinities for targeted C’ dots and free scFv fragments, with IC_50_ values of 305 nM and 107 nM, respectively, indicating that the conjugation of scFv fragments did not significantly alter binding affinity. Uptake of the targeted C’ dots in BT-474 tumor-bearing mice was evaluated through PET imaging, yielding an average value of 13.2% ID/g within 24–48 h p.i. (Figure 7B,C). Tumor uptake varied among animals, ranging from 10.3 to as high as 17.2% ID/g at 24 h p.i., likely reflecting differences in HER2 expression levels. By contrast, significantly lower tumor uptake values (≈5% ID/g) were observed in two separate controls, namely, non-targeted C’ dots in BT-474 tumors and targeted C’ dots in HER2-negative tumors (Figure 7B,C). Liver uptake was estimated at 8% ID/g at 2 h p.i., but this value fell to around 5% ID/g at 72 h p.i. Another interesting observation was the considerable tissue penetration and diffusion of targeted C’ dots observed in ex vivo BT-474 specimens through optical imaging and autoradiography (Figure 7D). Conversely, the C’ dots were mostly confined along the tumor periphery in both the two negative control groups.

Detappe et al. designed Gd-chelated polysiloxane NPs (AGuIX^®^) targeted with full-length antibodies against the B-cell maturation antigen (BCMA), which is almost exclusively present on the surfaces of mature B cells [149]. In addition, BCMA overexpression and activation are associated with multiple myeloma (MM), highlighting its potential as a therapeutic target for MM [161]. Based on this, the Gd construct was utilized for the detection of malignant plasma cells in MM using magnetic resonance imaging (MRI). Competitive cell-binding assays confirmed the maintenance of antibody specificity after conjugation to usNPs. The resulting targeted usNPs were 8–10 nm in size and exhibited efficient renal clearance, with over 90% ID found in the urine at 72 h p.i. Such a high clearance rate was unexpected considering the larger size of the construct compared to the kidney filtration barrier, and the underlying mechanism for this phenomenon requires further investigation. Quantification of Gd uptake in the tumor tissue at 30 min p.i. revealed 4.1% ID/g for targeted usNPs against 0.11% ID/g for the non-targeted particles. The rapid tumor uptake of the targeted usNPs, combined with their efficient renal clearance, resulted in an enhanced MRI signal-to-noise ratio for MM detection, surpassing levels achievable with other clinically approved imaging modalities. As early as 30 min post-administration, animals treated with the targeted usNPs demonstrated a ≈3-fold improvement in SNR for plasmacytomas in the spine.

Song et al. synthesized AuNCs protected by biocompatible cyclodextrin with a core size of 1.85 nm [147]. These particles exhibited strong photoluminescence upon excitation at 808 nm, with an emission peak at 1050 nm. Photoluminescence imaging in the second NIR window (1000 to 1700 nm) is advantageous for in vivo deep-tissue imaging as it experiences reduced interference from autofluorescence and photon scattering in tissues. The authors further conjugated the AuNCs with an antibody against the epithelial cell adhesion molecule (CD326), a well-known marker for various human carcinomas. Conjugation was achieved through host–guest complexation between cyclodextrin and adamantyl-modified anti-CD326 antibody. Subsequently, breast tumor-bearing mice were intravenously injected with antibody-targeted and non-targeted control AuNCs. Both AuNCs were efficiently excreted through the urine, reaching 75% ID at 24 h p.i. It was further observed that tumor accumulation of the targeted AuNCs reached ≈12% ID, marking a fourfold increase over the control group. Photoluminescence imaging effectively visualized the tumor sites in mice, yielding a ≈3-fold enhancement in emission signal intensity relative to the control animals.

### 6 Clinical applications of targeted ultrasmall NPs

Since the introduction of liposomal doxorubicin in 1995, there has been a significant research effort in the field of nanomedicine-based drugs [162]. Yet, only a select few have successfully progressed to the market, highlighting the challenges inherent in this developmental process. Notably, usNPs offer the potential to overcome many limitations associated with more traditional larger particles. Their efficacy has been validated in preclinical models, and several usNP formulations based on the AGuIX and C’ dot designs are currently undergoing clinical trials [163–168]. The prevalence of silica-based NPs in these trials is justified by their FDA approval as an investigational new drug for oncologic applications since 2011. Table 3 lists the usNPs in clinical development, as found on the website https://ClinicalTrials.gov.

**Table 3:** Survey of renal clearable ultrasmall NPs investigated in clinical trials. Both passive and active targeting systems are included.

Particle	Targeting strategy	Condition/disease	Main application	Status

AGuIX	passive	cervical cancer	MRI radiotherapy	NCT03308604 phase 1
AGuIX	passive	multiple brain metastases	MRI radiotherapy	NCT02820454 phase 1 (completed)
AGuIX	passive	glioblastoma	MRI radiotherapy	NCT04881032 phase 1/Phase 2
AGuIX	passive	multiple brain metastases	MRI radiotherapy	NCT03818386 phase 2
AGuIX	passive	brain metastases	MRI radiotherapy	NCT04094077 phase 2 (terminated)
AGuIX	passive	brain metastases	MRI radiotherapy	NCT04899908 phase 2
AGuIX	passive	pancreatic and lung tumors	MRI radiotherapy	NCT04789486 phase 1/phase 2
AGuIX	passive	various recurrent tumors	MRI proton therapy	NCT04784221 phase 2
cRGDY-PEG-Cy5.5-C’ dots	cRGDY	head and neck melanoma	optical imaging	NCT02106598 phase 1/phase 2
^89^Zr-DFO-cRGDY-PEG-Cy5-C’ dots	cRGDY	malignant brain tumors	PET imaging	NCT03465618 phase 1
^124^I-cRGDY-PEG-C’ dots	cRGDY	melanoma and brain tumors	PET imaging	NCT01266096 phase: not applicable
^64^Cu-NOTA-PSMA-PEG-Cy5.5-C’ dots	PSMA binding peptide	prostate cancer	PET/MR imaging	NCT04167969 phase 1

## Conclusion

Ultrasmall NPs represent a unique class of nanostructures for cancer nanomedicine, offering the potential to address significant limitations associated with larger particles, particularly in treating solid tumors.

The incorporation of active targeting ligands onto usNPs holds the potential to enhance their performance in cancer treatment, mainly by virtue of improving usNP retention and accumulation within the tumor tissue. Indeed, a multitude of preclinical studies have consistently shown that actively targeted usNPs exhibit increased tumor accumulation and improved outcomes in both therapeutic and diagnostic applications, surpassing the performance of non-targeted counterparts. Importantly, the incorporation of targeting ligands onto the surface of usNPs does not compromise their ability to undergo renal clearance or to evade recognition by the RES system.

Ultrasmall NPs have successfully entered human clinical trials. The website https://ClinicalTrials.gov lists twelve clinical trials utilizing silica-based usNPs as imaging and/or therapeutic agents, with four of these trials incorporating active targeting strategies. However, the extent to which active targeting will demonstrate similar beneficial outcomes in humans as observed in preclinical studies remains to be determined.

As actively targeted usNPs share similar physicochemical and biological characteristics with proteins, they may be anticipated to exhibit more favorable and predictable behavior in vivo compared to their larger counterparts. To further optimize the in vivo performance of actively targeted usNPs, it will be essential to gather comprehensive and quantitative insights into various factors, including (i) characterizing usNP–receptor binding affinity and kinetics; (ii) understanding usNP blood clearance, urinary excretion, and uptake by RES organs; (iii) investigating usNP long-term tissue accumulation and potential toxicity; (iv) quantifying usNP tumor extravasation and intravasation; and (v) assessing usNP intratumoral diffusion. Integrating this knowledge will enable mathematical modeling of body clearance, tumor uptake, and intratumoral distribution of usNPs, aiding the design of second-generation targeted usNPs displaying improved tumor targeting, therapeutic efficacy, and safety.

## Data Availability

Data sharing is not applicable as no new data was generated or analyzed in this study.
